# SAP97-mediated ADAM10 trafficking from Golgi outposts depends on PKC phosphorylation

**DOI:** 10.1038/cddis.2014.492

**Published:** 2014-11-27

**Authors:** C Saraceno, E Marcello, D Di Marino, B Borroni, S Claeysen, J Perroy, A Padovani, A Tramontano, F Gardoni, M Di Luca

**Affiliations:** 1 Department of Pharmacological and Biomolecular Sciences, Centre of Excellence on Neurodegenerative Diseases, Università degli Studi di Milano, via Balzaretti 9, 20133 Milan, Italy; 2 Department of Physics, Sapienza University of Rome, P.le A. Moro, 5-00187 Rome, Italy; 3 Department of Neurological Sciences, University of Brescia, 25125 Brescia, Italy; 4 CNRS, UMR-5203, Institut de Génomique Fonctionnelle, Montpellier, France; 5 Inserm, U661, Montpellier, France; 6 Universités de Montpellier 1 and 2, UMR-5203, Montpellier, France; 7 Institute Pasteur Fondazione Cenci Bolognetti, Sapienza University of Rome, P.le A. Moro, 5-00187 Rome, Italy

## Abstract

A disintegrin and metalloproteinase 10 (ADAM10) is the major *α*-secretase that catalyzes the amyloid precursor protein (APP) ectodomain shedding in the brain and prevents amyloid formation. Its activity depends on correct intracellular trafficking and on synaptic membrane insertion. Here, we describe that in hippocampal neurons the synapse-associated protein-97 (SAP97), an excitatory synapse scaffolding element, governs ADAM10 trafficking from dendritic Golgi outposts to synaptic membranes. This process is mediated by a previously uncharacterized protein kinase C phosphosite in SAP97 SRC homology 3 domain that modulates SAP97 association with ADAM10. Such mechanism is essential for ADAM10 trafficking from the Golgi outposts to the synapse, but does not affect ADAM10 transport from the endoplasmic reticulum. Notably, this process is altered in Alzheimer's disease brains. These results help in understanding the mechanism responsible for the modulation of ADAM10 intracellular path, and can constitute an innovative therapeutic strategy to finely tune ADAM10 shedding activity towards APP.

Genetic studies on Alzheimer's disease (AD) point to risk factor genes encoding proteins with a known function in local trafficking.^1^ With different approaches, the intracellular transport of a disintegrin and metalloproteinase 10 (ADAM10), the enzyme responsible for the *α*-secretase cleavage preventing the formation of amyloid *β* in primary neurons,^2, 3^ has also been described. ADAM10 contains an endoplasmic reticulum (ER) retention signal,^4^ whereas its activity is mainly localized in the *trans*-Golgi network or at the plasma membrane.^2, 5^

We have previously identified synapse-associated protein-97 (SAP97), a member of the membrane-associated guanylate kinase family of protein scaffolds that govern the trafficking of glutamate receptors, as an ADAM10 partner. SAP97 binds to the proline-rich sequences of the ADAM10 cytosolic domain with its SRC homology 3 (SH3) domain, thereby driving the protease to the postsynaptic membrane and increasing *α*-secretase cleavage.^6^ Interestingly, the ADAM10/SAP97 interaction is reduced in the hippocampus of AD patients^7^ and the disruption of ADAM10/SAP97 association in rodents leads to the generation of a non-transgenic model of the disease.^8^ On the other hand, ADAM10 membrane retrieval is mediated by an AP2-clathrin-dependent mechanism implicated in the dynamic regulation of ADAM10 synaptic localization/activity.^9^ All these data claim for a role of ADAM10 trafficking in the pathogenesis of AD.

Despite this knowledge, the intracellular signaling pathways regulating ADAM10 trafficking are still explored limitedly. Few studies independently reported that protein kinase C (PKC) and mitogen-activated protein kinase constitute two central signaling hubs for the regulation of *α*-secretase cleavage.^10^

In particular, the activation of PKC fosters the non-amyloidogenic *α*-secretase cleavage pathway,^11, 12, 13, 14^ and treatment with a PKC activator increases ADAM10 substrates' cleavage.^15^ In addition, the capability of PKC of regulating ADAM10 activity may be related to a modification of ADAM10 subcellular localization.^15^

Here, we identified a PKC phosphorylation site in the SH3 domain of SAP97 able to modulate the interaction with ADAM10 and promoting its trafficking from dendritic Golgi outposts to the synapse. These results contributed to the understanding of the mechanism responsible for the modulation of ADAM10 intracellular path, and could provide the background for the development of effective therapeutic strategy to tune ADAM10 activity.

## Results

### PKC activation fosters ADAM10/SAP97 interaction

**Figure 1:**
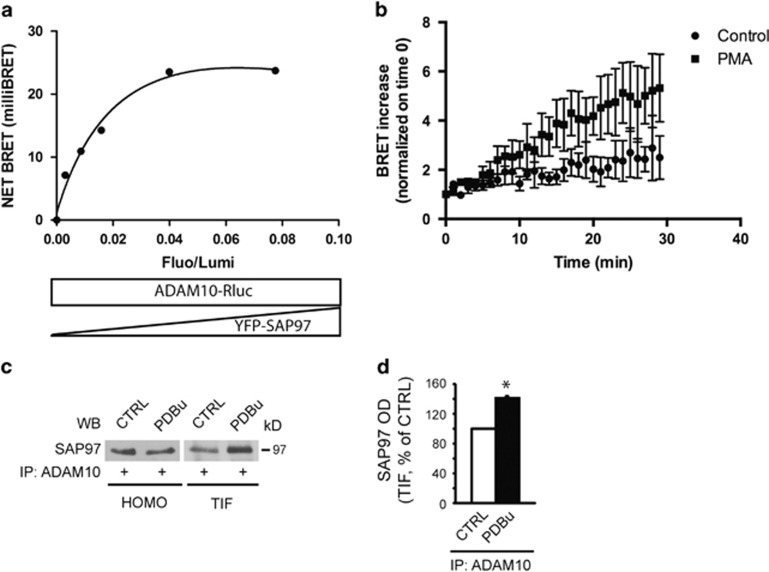
PKC activation promotes ADAM10/SAP97 association. (**a**) BRET experiments in living human embryonic kidney 293 (HEK293) cells. We fused the C terminus of ADAM10 to the energy donor *Renilla* luciferase (ADAM10-Rluc) and the N terminus of SAP97 to the acceptor YFP (YFP-SAP97). Under the condition of a constant level of ADAM10-Rluc expression, BRET signal increased hyperbolically as a function of YFP-SAP97 expression level. Saturation of the BRET signal when all the donor molecules were linked to the acceptor indicated a specific interaction between ADAM10 and SAP97 proteins (*R*^2^=0.9824, nonlinear regression equation, assuming a single binding site; GraphPad Prism, La Jolla, CA, USA). (**b**) Phorbol 12-myristate 13-acetate (PMA) treatment increases BRET signal after 30 min in cells transfected with ADAM10-Rluc and YFP-SAP97 (Fluo/Lumi=0.091±0.009). Data represent mean±S.E.M. of three independent experiments. (**c**) Total homogenates (HOMO) and TIF of control (CTRL) and PDBu-treated hippocampal slices were immunoprecipitated (IP) with a pAb to ADAM10 and SAP97 co-precipitation was evaluated. PDBu increases ADAM10/SAP97 co-precipitation only in TIF but not in HOMO. (**d**) Quantification of experiments in (**c**) (*n*=3, * *P*=0.006, PDBu *versus* CTRL, paired *t*-test). In this and all subsequent figures, data represent mean±S.E.M.

We first verified the effect of PKC activation on ADAM10/SAP97 interaction by bioluminescence resonance energy transfer (BRET) experiments. After validating the methodology (Figure 1a), the real-time BRET analysis showed that PKC activation induces an upward trend of BRET signal after the application of a PKC activator (Figure 1b).

Co-immunoprecipitation (IP) assays from hippocampal slices revealed a significant increase in ADAM10/SAP97 binding 30 min after PKC activation and showed that it occurs in a specific compartment, that is, the Triton-insoluble fraction (TIF), a synaptic and cellular membrane subfraction ^16^ (Figures 1c and d).

### PKC phosphorylation of SAP97 T629 promotes ADAM10/SAP97 interaction

**Figure 2:**
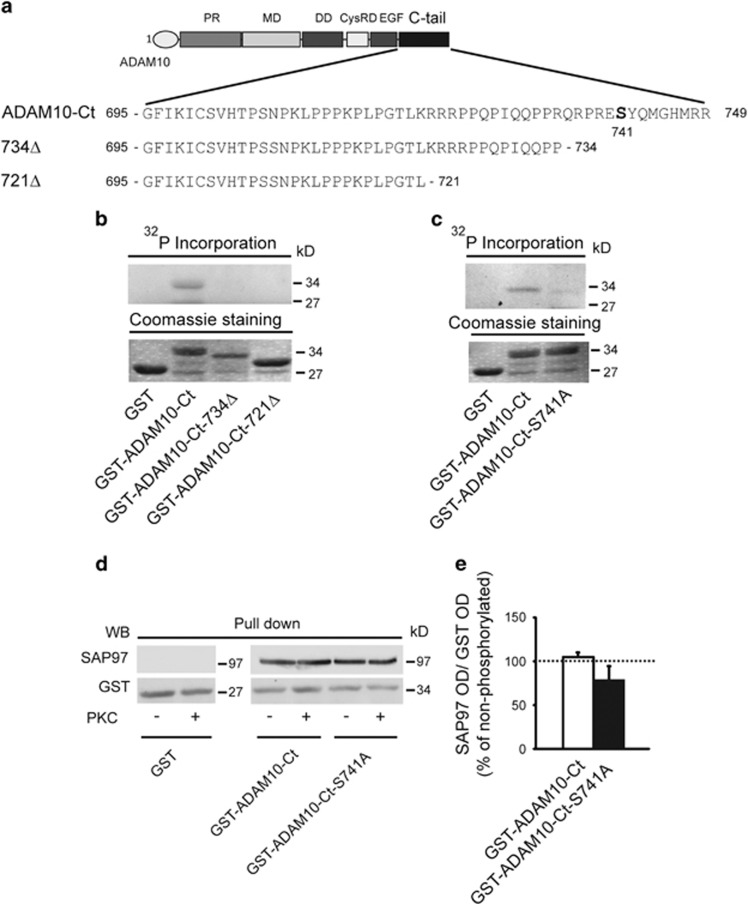
PKC-dependent phosphorylation of ADAM10 tail does not influence the association to SAP97. (**a**) ADAM10 cytoplasmic tail (Ct) and deletion mutants of aa sequences. In bold is the putative PKC phosphosite. (**b**) GST-ADAM10-Ct and deletion mutants were incubated or not with PKC in the presence of [*γ*-^32^P]ATP. Only GST-ADAM10-Ct is phosphorylated. Fusion proteins levels were comparable as shown by Coomassie staining (lower panel). (**c**) *In vitro* phosphorylation of GST-ADAM10-S741A fusion protein. The mutation S741A leads to a strong reduction of ADAM10-Ct phosphorylation. Fusion protein levels were comparable as shown by Coomassie staining (lower panel). (**d**) After PKC phosphorylation, GST fusion proteins of ADAM10 tail were used to perform pull-down assays to detect SAP97. PKC phosphorylation of ADAM10 does not affect SAP97 binding. (**e**) Quantification of experiments in (**d**) (*n*=3, *P*>0.05 phosphorylated *versus* non-phosphorylated, paired *t*-test). In this and all subsequent pull-down experiments, data were normalized on GST optical density (OD) and shown as the percentage of non-phosphorylated proteins in the same experiment

We next assessed whether either ADAM10 or SAP97 is a PKC substrate. First, the amino-acid (aa) analysis revealed S741 as a putative PKC phosphorylation site in the ADAM10 domain interacting with SAP97^6^ (Figure 2a).^17^ *In vitro* phosphorylation assays revealed PKC-dependent incorporation of phosphorus-32 (^32^P) only into the wild-type (wt) ADAM10-C-terminal (Ct) domain. Both the sequential deletion mutants of ADAM10 tail and the S741 to A mutant failed to show ^32^P incorporation, thus confirming that S741 is a PKC phosphorylation site (Figures 2b and c). To assess the role of the identified PKC phosphorylation site in the modulation of ADAM10/SAP97 interaction, we carried out pull-down assays with glutathione *S*-transferase (GST) fusion proteins phosphorylated or not by PKC. Neither the phosphorylation of ADAM10-Ct nor the mutation into A of the PKC phosphosite S741 affects ADAM10 binding to SAP97 (Figures 2d and e), suggesting that it is not relevant for the formation of the ADAM10/SAP97 complex.

**Figure 3:**
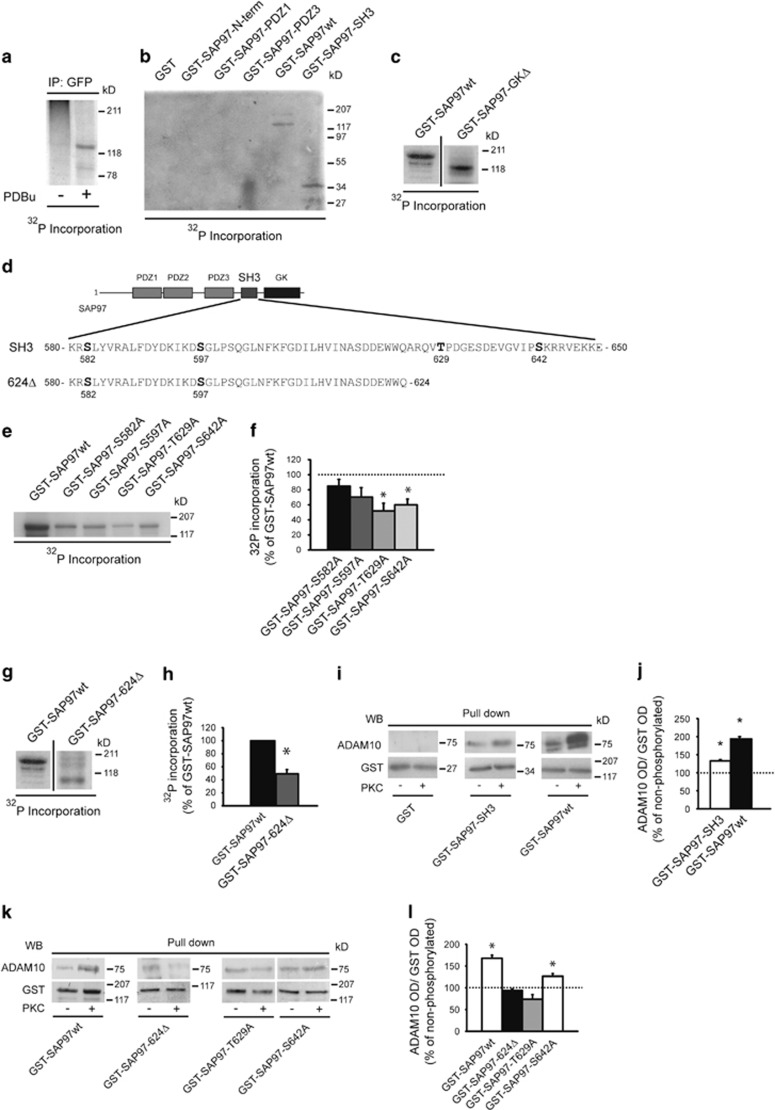
PKC phosphorylation of SAP97 T629 triggers ADAM10/SAP97 association. (**a**) COS-7 cells transfected with YFP-SAP97wt were incubated with [^32^P]orthophosphate and then exposed or not to PDBu. Cell lysates were IP with anti-GFP antibody (Ab). Autoradiography revealed a specific radioactive band corresponding to YFP-SAP97 in PDBu-treated cells. (**b**) *In vitro* phosphorylation of GST-SAP97wt and GST fusion proteins of different SAP97 domains. Autoradiography revealed two specific bands corresponding to GST-SAP97wt and GST-SAP97-SH3. (**c**) *In vitro* phosphorylation of GST-SAP97wt and GST-SAP97-GKΔ. Proteins were separated by sodium dodecyl sulfate-polyacrylamide gel electrophoresis (SDS-PAGE) and revealed by autoradiography. Samples derive from the same experiment and gel, but the lanes were non-adjacent in the gel. The lack of SAP97 HOOK-GK domains does not affect PKC phosphorylation of SAP97 (*n*=3, GST-SAP97-GKΔ=+0.4±7.7% *P*>0.05, GST-SAP97-GKΔ *versus* GST-SAP97wt, paired *t*-test). (**d**) SAP97-SH3 domain and SAP97-624Δ deletion mutant aa sequences. In bold is the putative PKC phosphosites. (**e**) *In vitro* phosphorylation of GST-SAP97 fusion proteins carrying single mutations of the putative PKC phosphosites. Only GST-SAP97-T629A and GST-SAP97-S642A mutants show a decrease in PKC phosphorylation compared with GST-SAP97wt. (**f**) Quantification of ^32^P incorporation of experiments in (**e**) (*n*=3, *P=0.0096 GST-SAP97-T629A *versus* GST-SAP97wt; *P*=0.0063 GST-SAP97-S642A *versus* GST-SAP97wt, unpaired *t*-test). (**g**) *In vitro* phosphorylation of GST-SAP97wt and GST-SAP97-624Δ. The lack of T629 and S642 significantly decreases PKC phosphorylation of SAP97. Samples derive from the same experiment and gel, but the lanes were non-adjacent in the gel. (**h**) Quantification of ^32^P incorporation of the experiments in (**g**) (*n*=3, *P<0.05 GST-SAP97-624Δ *versus* GST-SAP97wt, paired *t*-test). Data are expressed as the percentage of GST-SAP97wt. (**i**) GST-SAP97wt and GST-SAP97-SH3 were either or not *in vitro* phosphorylated by PKC and then incubated with rat brain HOMO to precipitate ADAM10. PKC phosphorylation increases ADAM10 binding. (**j**) Quantification of experiments in (**I**) (*n*=3, *P=0.01 GST-SAP97-SH3 phosphorylated *versus* non-phosphorylated; *P*=0.0026 GST-SAP97wt phosphorylated *versus* non-phosphorylated, paired *t*-test). (**k**) Pull-down analysis of ADAM10 binding to different mutants of GST-SAP97 after *in vitro* PKC phosphorylation. The lack of T629 prevents the PKC-induced increase in ADAM10/SAP97 association. (**l**) Quantification of experiments in (**k**) (*n*=3, *P=0.011 GST-SAP97wt phosphorylated *versus* non-phosphorylated; *P*=0.0475 GST-SAP97-S642A phosphorylated *versus* non-phosphorylated, paired *t*-test)

In a second step, we investigated whether SAP97 is a PKC substrate, carrying out metabolic labeling experiments with [^32^P]orthophosphate in yellow fluorescent protein (YFP)-SAP97wt-transfected COS-7 cells. Autoradiography revealed a specific radioactive band corresponding to YFP-SAP97 in phorbol 12,13-dibutyrate (PDBu)-treated cells samples, showing that SAP97 undergoes PKC phosphorylation in living cells (Figure 3a). We tested the presence of PKC phosphorylation sites within different SAP97 domains. GST-SAP97wt and GST-SAP97-SH3 fusion proteins, but not the other deletion mutants, are phosphorylated (Figure 3b). Moreover, the deletion mutant GST-SAP97-GKΔ is phosphorylated to the same extent as SAP97wt, demonstrating the absence of relevant PKC phosphosites in the HOOK-GK domain (Figure 3c). The analysis of the SH3 sequence predicted four PKC consensus motifs (S582, S597, T629 and S642; Figure 3d). *In vitro* phosphorylation assays with GST-SAP97wt or S/T to A point mutants at the putative PKC phosphosites showed that both mutations T629 and S642 to A reduced SAP97 phosphorylation, whereas the replacement of S582 and S597 did not significantly affect ^32^P incorporation (Figures 3e and f). Consistently, the loss of both T629 and S642 in the GST-SAP97-624Δ deletion mutant lacking the Ct region of SAP97 significantly decreased ^32^P incorporation (Figures 3d, g and h). To evaluate whether PKC phosphorylation of SAP97 modifies ADAM10/SAP97 interaction, we carried out pull-down assays with GST fusion proteins phosphorylated or not by PKC. The analysis revealed that the phosphorylation of both GST-SAP97wt and GST-SAP97-SH3 significantly increases the association with ADAM10 (Figures 3i and j).

Notably, the elimination of both phosphosites in the deletion mutant GST-SAP97-624Δ abolishes the PKC-induced increase in ADAM10 precipitation (Figures 3k and l). However, the mutation of T629 to A, but not the mutation of S642 to A, prevented the PKC-induced increase in ADAM10/SAP97 association, thus indicating that only the T629 phosphorylation site is relevant for the ADAM10/SAP97 interaction (Figures 3k and l). Finally, neither the mutation of the other putative PKC phosphosites nor the HOOK-GK domain deletion influences the PKC-induced ADAM10 binding to SAP97 (Supplementary Figures 1A and B).

Supporting for specificity, PKC phosphorylation of GST-SAP97wt and of the single mutants lacking SH3 phosphosites did not alter the binding to other SAP97 partners, that is A-kinase anchor protein 150 (AKAP150), GluA1 and GluN2A (Supplementary Figures 1C–E).

These results demonstrate that SAP97 phosphorylation at T629 positively modulates ADAM10/SAP97 interaction.

### Phosphorylation of T629 influences SAP97 structure

To explore further how SAP97 T629 phosphorylation influences ADAM10/SAP97 binding, we carried out a modeling study.

**Figure 4:**
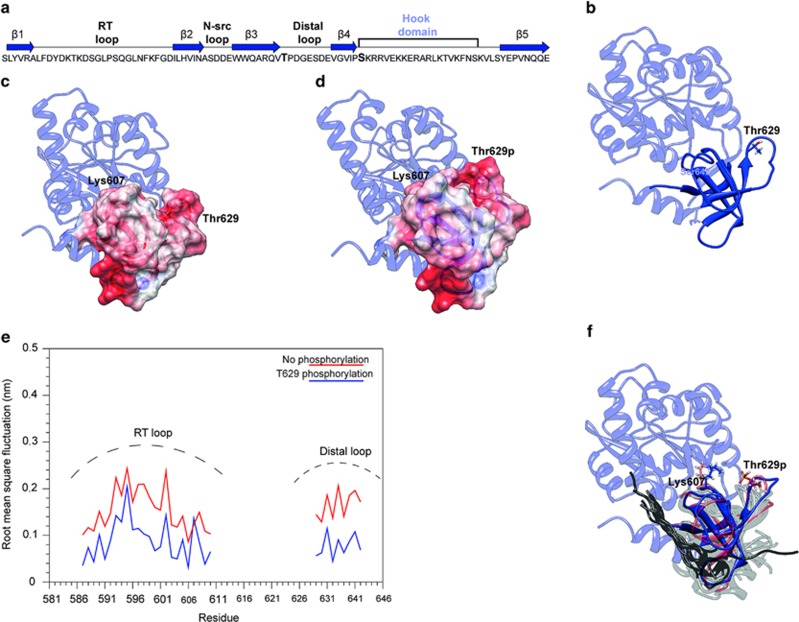
PKC phosphorylation of the T629 residue affects SAP97 structure. (**a**) Aa sequence and topology of the SH3 domain of SAP97. The blue arrows represent the *β*-strands and the lines represent the loops. The HOOK domain is also indicated. Residues T629 and S642 are shown in bold. (**b**) Ribbon representation of the SH3-HOOK-GK domains of SAP97. The SH3 domain is shown in blue and the HOOK and GK domains in light blue. Residues T629 and S642 are shown as blue sticks. (**c** and **d**) Electrostatic potential mapped on the molecular surface of the SH3 domain in the absence (**c**) or presence (**d**) of the phosphorylation on residue T629. The HOOK and GK domains are shown in light blue, whereas the SH3 domain is colored according to the electrostatic potential, blue is positive, white is neutral and red is negative. Residues K607 and T629 are shown as sticks. (**e**) The RMSF of the RT and distal loops. Per-residue RMSF of the RT and distal loops in the presence (blue line) and in the absence (red line) of the T629 phosphorylation. (**f**) Ribbon representation of the SH3 domain of SAP97 extracted from the simulation in the absence (red) or presence (blue) of the phosphorylation. The remaining of the protein is in light blue. The SH3 domains superimposed with the SAP97-SH3 domain are shown in light grey, and the cocrystallized peptides in dark grey. Residues T629 and K607 are shown as sticks

The 3D structure of the SH3-GK domains of the SAP97 protein was recently solved by x-ray crystallography.^18^ The SH3 domain of SAP97 is formed by five *β*-strands and has been defined as a ‘splitted' SH3,^18^ because of a peculiar feature: strand *β*5 is located after an intervening domain called HOOK domain and not contiguous in sequence with the other four strands (Figure 4a).^18^ T629 is located at the beginning of the so-called ‘distal loop' that connects strands *β*3 and *β*4, whereas S642 is found at the beginning of the HOOK domain (Figures 4a and b).

The phosphorylated site T629 was modeled in the 3D structure of the SAP97-SH3 domain subsequently energy minimized. As expected, the introduction of the charged phosphate group causes a change in the electrostatic potential (Figures 4c and d). The major differences in the electrostatic potential are located in the RT and distal loops of the SAP97-SH3 domain (Figures 4c and d). Inspection of the minimized structure suggests that the phosphorylation allows the formation of an electrostatic interaction of the phosphate with K607 located on the RT loop, an interaction that cannot be established in the absence of phosphorylation (Figures 4c and d). This is expected to increase the rigidity of the RT loop, involved in the interaction with other molecular partners, as ADAM10.

To verify this hypothesis, molecular dynamics simulations of 10 ns each were carried out for the SAP97-SH3 complex in the presence and absence of the T629 phosphorylation. Figure 4e shows the root mean square fluctuation (RMSF) calculated for the RT and distal loop in the presence and absence of the T629 phosphorylation. In the absence of the phosphate group, the two loops are mobile and assume different conformations increasing the structural flexibility of the interaction site (Figure 4e). Upon phosphorylation, the structure of the SH3 domain becomes more rigid, in particular at the level of the RT and distal loops that are locked in a defined conformation (Figures 4e and f). The rigidity of these loops affects the structure of the binding site specific for the PxxP motif.

Figure 4f shows the structures of 15 different SH3 domains cocrystallized with different partners and superimposed to the snapshots of Figures 4a and b. All the peptides cocrystallized with the SH3 domains are in direct contact with strands *β*3 and *β*4 and with the RT loop (Figure 4f), which, therefore, potentially represents the binding site for ADAM10.

Considering these results, the phosphorylation of T629 is expected to mainly affect the RT loop. As a consequence, the SAP97 binding site assumes a more rigid conformation leading to a stronger interaction with the Ct region of ADAM10.

### The phosphorylation of SAP97 at T629 has a functional and pathological role

ADAM10/SAP97 interaction is reduced in AD patients' hippocampi at Braak 4, suggesting a SAP97 cargo dysfunction in the pathology.^7^ Therefore, we asked whether an alteration of PKC phosphorylation at the residue T629 of SAP97 could be responsible for the decrease in ADAM10/SAP97 association in AD patients.

To this, a phospho-specific antibody (Ab) raised against T629 (SAP97-T629P Ab) was produced, affinity purified and tested for specificity (Supplementary Figures 2A and B).

**Figure 5:**
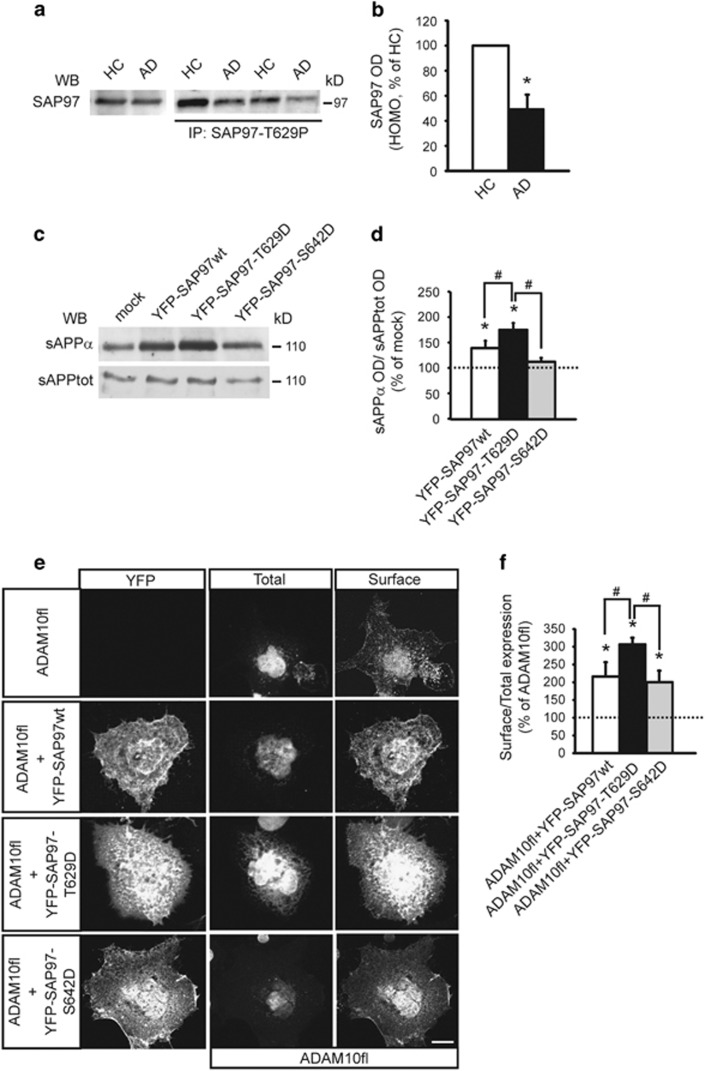
The phosphorylation of SAP97 at T629 is reduced in AD patients' hippocampi and affects ADAM10 localization/activity. (**a**) IP carried out from HCs and AD patients post-mortem hippocampi homogenates (HOMO) with an Ab raised against phosphorylated T629 (anti-SAP97-T629P). WB analysis was performed with SAP97 Ab. SAP97 T629 phosphorylation is reduced in AD patients compared with HCs. Leftmost lanes, WB performed on HCs and AD hippocampi HOMO showed no alterations of SAP97 levels. (**b**) Quantification of IP experiments in (**a**) (*n*=12, *P=0.012 AD *versus* HCs, paired *t*-test). (**c**) Representative WB of sAPPα and total sAPP released by human embryonic kidney 293 (HEK293) cells stably expressing APP and transfected with either YFP-SAP97wt or phosphomimetic mutants. SAP97wt expression stimulates ADAM10 activity, and the mutant mimicking T629 phosphorylation further increases this effect. (**d**) Quantitative analysis of experiments in (**c**). The levels of sAPPα were normalized on total sAPP and data are expressed as the percentage of mock cells (*n*=4, *P<0.05 *versus* mock; ^#^*P*<0.05 *versus* YFP-SAP97-T629D, one-way analysis of variance (ANOVA), Bonferroni's *post hoc* test). (**e**) COS-7 cells were transfected with ADAM10 full-length (fl) alone or co-transfected with either YFP-SAP97wt or phosphomimetic mutants and stained for ADAM10 surface/total expression. ADAM10fl alone was faintly localized at the surface despite intense intracellular labeling. In contrast, the co-transfection of ADAM10fl and YFP-SAP97wt or the mutants increased ADAM10fl surface expression. Notably, the YFP-SAP97-T629D mutant had the strongest effect on ADAM10fl surface expression. Scale bar, 10 μm. (**f**) Quantification of experiments in (**e**) (*P<0.05 *versus* ADAM10fl; ^#^*P*<0.05 *versus* YFP-SAP97-T629D; 16 cells per condition from two independent experiments; one-way ANOVA, Bonferroni's *post hoc* test)

We analyzed the levels of SAP97 phosphorylation at T629 in hippocampi from AD patients at Braak 4^19^ and from age-matched healthy controls (HCs). Total homogenates from hippocampi of HCs and AD patients were IP with SAP97-T629P Ab and total SAP97 was evaluated. Quantitative analysis showed a significant reduction in SAP97 phosphorylation in AD hippocampi when compared with HCs (Figures 5a and b). The IP was specific as no signal was detected in the absence of the phospho-Ab (Supplementary Figure 2C). As previously demonstrated, SAP97 total levels were not affected by the sample group.^7^

To verify whether genetic modulation of ADAM10/SAP97 interaction might occur, 100 AD patients^20^ were recruited, and genetic polymorphism within the SH3 domain of SAP97 evaluated. No genetic variations within the SH3 domain of SAP97 were detected in AD patients, suggesting that other cellular mechanisms might be responsible for the decreased PKC phosphorylation of SAP97 at T629 in AD.

Because SAP97 regulates ADAM10 synaptic membrane localization and activity,^6^ we tested whether this specific phosphorylation could have a role in controlling ADAM10 activity. Soluble amyloid precursor protein α (sAPPα) release was significantly increased when HEK293 cells stably expressing human APP were transfected with YFP-SAP97wt. However, sAPPα levels further increased when cells expressed a mutant mimicking the phosphorylation of SAP97 at T629 (YFP-SAP97-T629D), but not at S642 (YFP-SAP97-S642D), indicating that T629 phosphorylation specifically boosts ADAM10 activity (Figures 5c and d).

Further, COS-7 cells were transfected with ADAM10 alone or co-transfected with either YFP-SAP97wt or the phosphomimetic mutants. Coexpression of SAP97 yields a nearly twofold increase in ADAM10 surface levels. Yet, cells expressing SAP97 mutated in T629, but not in S642, display a stronger ADAM10 surface labeling when compared with SAP97wt-transfected cells. Quantitative analysis confirmed that SAP97 phosphorylation at T629, but not at S642, significantly increases ADAM10 surface/total levels (Figures 5e and f).

These data indicate a functional role of the PKC-dependent SAP97 phosphorylation at T629 in the regulation of ADAM10 localization/activity and an involvement of this process in AD.

### PKC activation induces SAP97-mediated ADAM10 trafficking from the Golgi outposts to the postsynaptic density

Based on these findings, we investigated how the PKC-dependent phosphorylation of SAP97 influences ADAM10 trafficking in neuronal cells.

To analyze ADAM10 and SAP97 distribution along the secretory pathway, we purified TIF and microsomal fraction (P3), enriched in the Golgi and ER proteins, from hippocampal slices (Supplementary Figure 2D). Quantitative analysis of IP assays revealed a significant increase in SAP97-T629P in the total homogenate (HOMO) and in the TIF, but not in the P3 fraction, in PDBu-treated slices when compared with control (Supplementary Figures 2E and F), thus suggesting that SAP97 phosphorylation at T629 mainly occurs in the synaptic membrane fraction, as the previously shown increase in ADAM10/SAP97 association (Figures 1c and d).

**Figure 6:**
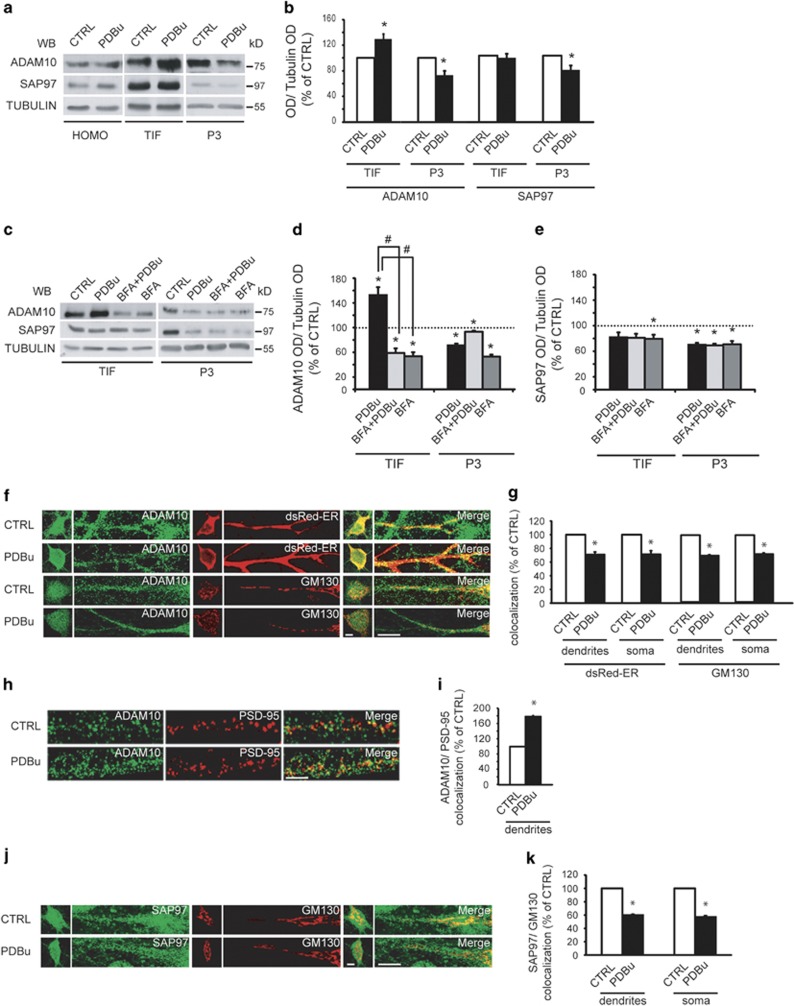
PKC activation promotes ADAM10 delivery to the postsynaptic compartment. (**a**) Representative WB of ADAM10 and SAP97 localization in HOMO, TIF and P3 fraction upon PKC activation in hippocampal slices (PDBu, 100 nM, 30 min). PKC activation modifies ADAM10 and SAP97 cellular distribution. (**b**) Quantification of experiments in (**a**) (*n*=7, ADAM10, *P=0.008, TIF, PDBu *versus* control (CTRL); *P*=0.008, P3, PDBu *versus* CTRL; SAP97, *P*=0.02, P3, PDBu *versus* CTRL, paired *t*-test). In this and all subsequent fractionation experiments, data are expressed as the percentage of CTRL. (**c**) Representative WB of ADAM10 and SAP97 localization in hippocampal slices treated with BFA before PKC activation. BFA affects ADAM10 but not SAP97 trafficking. (**d** and **e**) Quantification of experiments in (**c**) (*n*=5, *P<0.05 *versus* CTRL; ^#^*P*<0.05 *versus* PDBu, one-way analysis of variance (ANOVA), Bonferroni's *post hoc* test). (**f**) Staining of ADAM10/dsRed-ER and ADAM10/GM130 in dendrites and soma of neurons upon PDBu treatment. PKC activation decreases ADAM10 levels in dendritic and somatic ER and Golgi. In all experiments, primary neurons were kept in culture 14 DIV before treatment and staining experiments. Scale bar, 10 *μ*m. (**g**) Quantification of experiments in (**f**) (24 neurons per condition from three independent experiments, dsRED-ER: dendrites, *P=0.001; soma, *P=0.009; GM130: dendrites, *P=0.0017; soma, *P=0.0006, PDBu *versus* CTRL unpaired *t*-test). (**h**) ADAM10/PSD-95 staining showing that PKC activation increases ADAM10 levels in the PSD. Scale bar, 10 *μ*m. (**i**) Quantification of experiments in (**h**) (24 neurons per condition from three independent experiments, *P=0.00001, PDBu *versus* CTRL, unpaired *t*-test). (**j**) Representative staining of SAP97/GM130 in dendrites and soma of neurons after PDBu treatment; PKC activation reduces SAP97 levels in dendritic and somatic Golgi. Scale bar, 10 *μ*m. (**k**) Quantification of experiments in (**j**) (24 neurons per condition from three independent experiments, dendrites, *P=0.0001; soma, *P=0.0006, PDBu *versus* CTRL, unpaired *t*-test)

Furthermore, ADAM10 levels were increased in TIF and concomitantly reduced in the P3 fraction upon PDBu treatment, indicating that PKC activation triggers ADAM10 trafficking from ER and Golgi to the membrane. No alterations of total ADAM10 and SAP97 levels were detected (Figures 6a and b).

To determine which intracellular compartment is involved in PKC-induced ADAM10 trafficking, we used brefeldin-A (BFA), which inhibits the transport of proteins from ER to Golgi.^21^ Pre-treatment with BFA prevents the PKC-induced reduction of ADAM10 levels in the P3 fraction and the correspondent increase in TIF, whereas SAP97 trafficking was not affected (Figures 6c and e). Therefore, PKC activation triggers ADAM10 transport from the ER, whereas PKC-induced SAP97 trafficking involves post-ER compartments.

As these results indicate in principle the absence of a similar cellular distribution pattern of ADAM10 and SAP97 upon PKC activation, we carried out colocalization analyses in hippocampal cultures to clarify these data.

We transfected dsRed-ER to visualize both somatic ER and ER subcompartments.^22, 23^ *Cis*-Golgi marker 130 (GM130) immunoreactivity identified both somatic Golgi and dendritic Golgi outposts, whereas postsynaptic density-95 (PSD-95) staining revealed the postsynaptic density (PSD).

PKC activation significantly decreases both ADAM10/dsRed-ER and ADAM10/GM130 colocalization in dendrites and soma, and concomitantly increases ADAM10/PSD-95 colocalization (Figures 6f–i).

Conversely, PKC activation significantly reduces SAP97/GM130 colocalization in both dendrites and soma, without affecting SAP97 colocalization with both dsRed-ER and PSD-95 (Figures 6j and k and Supplementary Figures 3A and B).

These data indicate that PKC activation triggers ADAM10 transport from ER and Golgi, whereas PKC-induced SAP97 trafficking starts from the Golgi.

### PKC phosphorylation of SAP97 at T629 is relevant for ADAM10 local trafficking

To examine the subcellular localization of ADAM10/SAP97 complex formation, we used the previously characterized cell-penetrating Pro peptide, capable of interfering with the ADAM10/SAP97 interaction (Supplementary Figure 3C).^6, 8, 24^ Treatments with a control peptide (Ala) were used as control.

**Figure 7:**
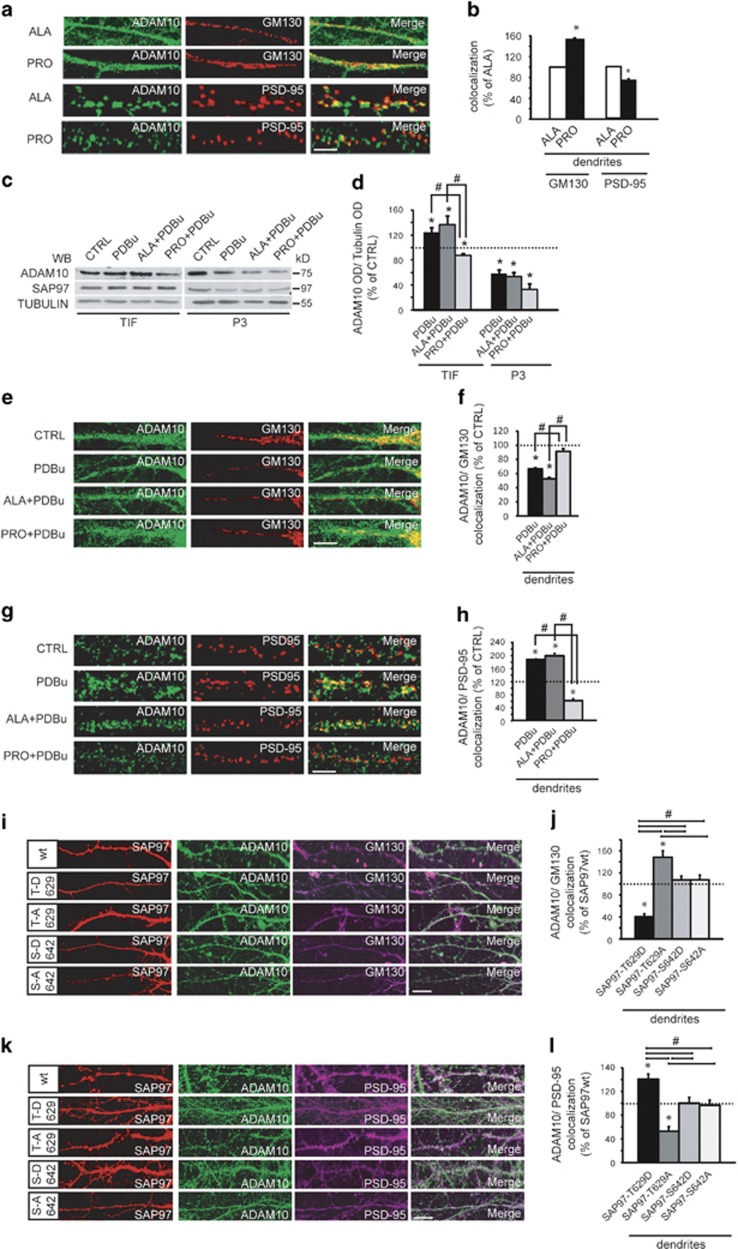
PKC phosphorylation of SAP97 at T629 triggers ADAM10 trafficking from Golgi outposts to the synapse. (**a**) ADAM10 and PSD-95/GM130 staining in dendrites of neurons after either Ala or Pro peptide treatment. Pro peptide affects ADAM10 distribution pattern in Golgi outposts and in the PSD. Scale bar, 10 *μ*m. (**b**) Quantification of experiments in (**a**) (24 neurons per condition from three independent experiments, GM130, *P=0.0006; PSD-95, *P=0.04, Pro *versus* Ala, unpaired *t*-test). Data are expressed as the percentage of Ala. (**c**) Representative WB of ADAM10 and SAP97 localization in hippocampal slices pre-treated with either Ala or Pro peptide and exposed to PDBu; Pro peptide prevents ADAM10 trafficking to the synapse without affecting SAP97 distribution. (**d**) Quantification of experiments in (**c**) (*n*=5, * *P*<0.05 *versus* CTRL; ^#^*P*<0.05 *versus* Pro+PDBu, one-way analysis of variance (ANOVA), Bonferroni's *post hoc* test). (**e**) ADAM10 localization in dendritic and somatic Golgi of either Ala peptide- or Pro peptide-pre-treated hippocampal neurons exposed to PDBu. Pro peptide prevents PDBu-induced ADAM10 trafficking from dendritic Golgi outposts. Scale bar, 10 *μ*m. (**f**) Quantification of experiments in (**e**) (24 neurons per condition from three independent experiments, *P<0.05 *versus* CTRL; ^#^*P*<0.05 *versus* Pro+PDBu, one-way ANOVA, Bonferroni's *post hoc* test). (**g**) ADAM10/PSD-95 staining in dendrites of neurons Ala/Pro treated before PDBu incubation. Pro treatment affects ADAM10 delivery to the PSD. Scale bar, 10 *μ*m. (**h**) Quantification of experiments in (**g**) (24 neurons per condition from three independent experiments, *P<0.05 *versus* CTRL; ^#^*P*<0.05 *versus* Pro+PDBu, one-way ANOVA, Bonferroni's *post hoc* test). (**i**) Representative dendritic ADAM10/GM130 staining of neurons transfected with either YFP-SAP97wt or phosphosite mutants. SAP97 mutants mimicking or abolishing T629 phosphorylation affect ADAM10 localization in Golgi outposts. Scale bar, 10 *μ*m. (**j**) Quantification of the experiments in (**I**) (16 neurons per condition from two independent experiments, *P<0.05 *versus* YFP-SAP97wt; ^#^*P*<0.05 *versus* YFP-SAP97-T629D or YFP-SAP97-T629A, one-way ANOVA, Bonferroni's *post hoc* test). (**k**) ADAM10/PSD-95 staining of neurons expressing either YFP-SAP97wt or phosphosites mutants. T629 mutations modifies ADAM10 synaptic levels. Scale bar, 10 *μ*m. (**l**) Quantification of experiments in (**k**) (16 neurons per condition from two independent experiments, *P<0.05 *versus* YFP-SAP97wt; ^#^*P*<0.05 *versus* YFP-SAP97-T629D or YFP-SAP97-T629A, one-way ANOVA, Bonferroni's *post hoc* test)

Pro peptide treatment in hippocampal neurons led to ADAM10 accumulation in Golgi outposts, as the enzyme failed to traffic to the postsynaptic compartment, as demonstrated by a decrease in the degree of ADAM10/PSD-95 colocalization (Figures 7a and b). No changes in ADAM10 localization in somatic Golgi and in somatic/dendritic ER (Supplementary Figures 3D and E) and no alterations in SAP97 distribution in ER, Golgi and PSD (Supplementary Figures 3F–H) were detected in Pro peptide-treated cells. These results indicate that ADAM10/SAP97 complex formation occurs in dendritic Golgi outposts, thus suggesting that SAP97-mediated ADAM10 trafficking follows a non-conventional secretory pathway.^23, 25^

We then reasoned that if SAP97 mediates the PKC-induced ADAM10 transport from Golgi outposts to the PSD, the uncoupling of ADAM10/SAP97 complex should prevent it.

To this, hippocampal slices were pre-treated with either Pro peptide or Ala peptide before PKC activation. Pro peptide treatment prevented the PKC-induced increase in ADAM10 levels in TIF but did not affect the reduction of ADAM10 in the P3 fraction (Figures 7c and d). As expected, the incubation with Pro peptide does not influence the PKC-triggered changes in SAP97 levels in the P3 fraction (Figure 7c).

These results were fully clarified by imaging experiments in neuronal cultures. The presence of the Pro peptide blocked PKC-induced ADAM10 trafficking from the dendritic Golgi towards the PSD, but did not affect PKC-triggered ADAM10 transport from somatic Golgi and from ER (Figures 7e and f and Supplementary Figures 4A and B). Moreover, the Pro peptide treatment did not interfere with PKC-induced changes in SAP97 cellular distribution (Supplementary Figures 4C–E). These data demonstrate that SAP97 is responsible for PKC-induced ADAM10 trafficking from dendritic Golgi outposts towards the PSD.

Are the effects of PKC activation on ADAM10 trafficking specifically due to the phosphorylation of SAP97 at T629? To address this question, we evaluated the effects of mutants either mimicking or abolishing the phosphorylation of SAP97 at T629 on ADAM10 localization.

Hippocampal neurons were transfected with either YFP-SAP97-T629D or YFP-SAP97-T629A and fixed to analyze ADAM10 colocalization with GM130 or PSD-95.

YFP-SAP97-T629D overexpression caused a significant decrease of ADAM10 levels in dendritic Golgi outposts and a correspondent increase in the percent overlap between dendritic ADAM10 and PSD-95 puncta. Conversely, in cells transfected with the mutant YFP-SAP97-T629A, ADAM10 accumulates in dendritic Golgi outposts and fails to traffic to the postsynaptic compartment. Furthermore, the transfection of the SAP97 mutants at S642, a PKC phosphosite not relevant to ADAM10/SAP97 association, does not influence ADAM10 localization in both dendritic Golgi and PSD, ruling out nonspecific effects (Figures 7i–l). No alterations of ADAM10 distribution in somatic Golgi are detectable (Supplementary Figure 4F).

## Discussion

Synaptic trafficking is a key modulator of *α*-secretase activity. However, signaling pathways able to foster local ADAM10 trafficking in spines are still unknown.

Here, we identify a novel PKC phosphosite in SAP97, the protein responsible for ADAM10 delivery to spines.^6^ PKC phosphorylates the SAP97-SH3 domain, modulating both ADAM10/SAP97 association and ADAM10 synaptic delivery. This mechanism has a pathogenic role, as we observed a significant reduction of SAP97 phosphorylation in AD patients, which can be responsible for the previously reported defect in ADAM10 synaptic trafficking and activity.^7^ Finally, we showed that ADAM10 trafficking implies transit through dendritic Golgi outposts where ADAM10/SAP97 complex formation occurs.

Our results demonstrate that PKC activation fosters ADAM10 forward trafficking towards the postsynapse inducing ADAM10 exit from the ER.^4^ We showed that PKC stimulation leads to a reduced ER/Golgi ADAM10 localization and to a concomitant increase in the enzyme levels in the PSD.

We have evaluated in parallel the localization of SAP97, previously found to be enriched in ER, where it has been proposed to convey AMPA (*α*-amino-3-hydroxy-5-methyl-4-isoxazolepropionic acid) receptors.^26^ Furthermore, SAP97 has been identified as a binding partner and substrate for PKC and is also required for PKC-dependent stimulation of cell migration.^27^ Imaging analysis revealed that PKC activation reduces SAP97 localization in both somatic Golgi and dendritic Golgi outposts.

Golgi outposts are considered to be an extension of the somatic Golgi^25^ or a site of biosynthesis for integral membrane proteins translated from dendritically localized mRNAs.^28, 29^ As SAP97 has been previously shown to be required for NMDARs trafficking via a non-conventional secretory pathway that uses dendritic Golgi outposts,^23^ we asked whether ADAM10/SAP97 complex also transits through this compartment.

When we interfere with ADAM10/SAP97 complex formation, using the cell-permeable Pro peptide,^6, 8^ ADAM10 fails to be delivered to the synapse and accumulates in dendritic Golgi outposts, suggesting that ADAM10 associates with SAP97 in this subcellular compartment.

Moreover, uncoupling ADAM10 and SAP97 prevents the PKC-triggered ADAM10 transport from Golgi outposts to the PSD without affecting enzyme sorting via the ER-somatic Golgi conventional path. Therefore, ADAM10/SAP97 interaction is necessary for the PKC-induced ADAM10 trafficking via the non-conventional secretory pathway, which may provide a platform for local control of ADAM10 insertion in the synapse, thus enabling neurons to more tightly and locally regulate its activity.

Furthermore, we found out that PKC activation positively modulates ADAM10 association to SAP97. Although it turned out that both ADAM10 and SAP97 are PKC substrates, only PKC phosphorylation of SAP97 affects ADAM10/SAP97 complex formation. We identified T629 within SAP97-SH3 domain as a novel PKC phosphosite, whose phosphorylation entails an increased interaction with ADAM10. Noteworthy, SAP97 T629 phosphorylation does not affect the interaction with other SAP97 binding partners, ruling out the chance that this posttranslational modification generally influences SAP97 binding capability. Molecular dynamics simulations demonstrated that T629 phosphorylation leads to a more rigid conformation of the SAP97 binding site and, thereby, is likely to mediate a stronger interaction with the ADAM10 cytoplasmic tail.

The phosphorylation of SAP97 T629 has important implications for ADAM10 activity because it affects ADAM10 membrane levels and, in turn, APP cleavage. In heterologous systems, the overexpression of the SAP97-T629D mutant, which mimics phosphorylation, fosters ADAM10 transport to the plasma membrane and increases sAPP*α* release. Then, imaging studies revealed that the phosphorylation of SAP97 T629 specifically promotes ADAM10 transport from Golgi outposts to the postsynaptic compartment. Conversely, the loss of this specific PKC phosphosite causes ADAM10 accumulation in Golgi outposts and decreases its synaptic levels.

We have previously demonstrated that the reduction of *α*-secretase activity is central in early stages of AD pathogenesis and is a consequence of a failure in ADAM10 exocytosis/endocytosis processes.^7, 9^ Here we describe a significant decrease in PKC-dependent phosphorylation of SAP97 T629 in AD patients' hippocampi, an outcome in line with previous studies revealing that AD patients have lower levels of PKC activity.^30^ Moreover, this alteration is not associated with the presence of genetic variations in the SH3 domain of SAP97.

These results add another tile to our understanding of the complex intracellular cascade of events responsible for the defective ADAM10 trafficking and APP cleavage in AD neurons. Whether ADAM10 forward trafficking and endocytosis are independent pathways or act synergistically to the development of defective APP cleavage still remains to be understood.

Recent studies demonstrated that reduction of *α*-secretase activity can cause AD.^31^ Moreover, a modest elevation of ADAM10 activity may be beneficial for AD and is well tolerated in adult brain, supporting the hypothesis that therapeutic strategies for increasing *α*-secretase activity via ADAM10 upregulation are predicted to be efficacious for AD.^31, 32, 33^

Hence, our findings provide a neuron-specific mechanistic framework according to which ADAM10 dendritic trafficking from Golgi outposts to the synapse and its shedding activity are under the control of the phosphorylation of SAP97 T629 by PKC. As this mechanism is altered in early stages of AD, it can represent a molecular target for brain-specific *α*-secretase-based therapy that could be a useful alternative or combination therapy with current therapeutics.

## Materials and Methods

### Human studies

Hippocampi from six AD patients and six HCs were obtained from the Netherland Brain Bank (NBB, Amsterdam, The Netherlands). Established Braak and Braak criteria were used to categorize AD tissues.^19^ AD patients fulfilled the Braak 4 stage. Accordingly, in AD cases there were tangles and neuritic plaques in the hippocampus. HCs have no history of psychiatric or neurological disease and no evidence of age-related neurodegeneration. Tissues were collected within a maximum post-mortem delay of 6–8 h to reduce degradation.

### TIF and P3 preparation

TIF and P3 were isolated from rat acute hippocampal slices. Slices were homogenized at 4 °C in an ice-cold lysis buffer with protease inhibitors, phosphatase inhibitors, 0.32 M sucrose, 1 mM HEPES, 0.1 mM PMSF, 1 mM MgCl_2_ using a glass-Teflon homogenizer (VWR, Radnor, PA, USA). HOMO was centrifuged at 10 000 × *g* for 20 min at 4 °C. The pellet (P1) was used to purify TIF and the supernatant (S1) to obtain P3 fraction. Triton X-100 extraction of the P1 was carried out at 4 °C for 20 min in an extraction buffer (0.5% Triton X-100, 150 mM KCl and protease inhibitors). After extraction, the samples were centrifuged at 100 000 × *g* for 1 h at 4 °C and the pellet (TIF) was resuspended in 20 mM HEPES with protease inhibitors. The Triton-soluble fraction was kept for western blot (WB) analysis. The supernatant S1 was centrifuged at 100 000 × *g* for 2 h at 4 °C and the microsomal pellet (P3) was resuspended in the lysis buffer.^34^

### Immunoprecipitation

Either aliquots of human hippocampi HOMO or HOMO/TIF/P3 fraction from hippocampal slices were incubated overnight at 4 °C in RIA buffer (200 mM NaCl, 10 mM EDTA, 10 mM Na_2_HPO_4_, 0.5% NP-40, 0.1% SDS) in a final volume of 150 μl with an Ab against ADAM10 or SAP97-T629P Ab. Protein A/G beads (Tebu-bio, Peterborough, UK) were added and incubation was continued for 2 h by shaking at RT. Beads were collected by centrifugation and washed three times with RIA buffer. In IP assays performed with SAP97-T629P Ab, the samples were incubated and washed with RIA buffer containing SDS 1% to avoid any interference of protein–protein interactions. Sample buffer for SDS-PAGE was added and the mixture was heated for 3 min. Beads were collected by centrifugation and a volume of supernatants was applied onto SDS-PAGE; the immunocomplex precipitated was revealed by anti-SAP97 Ab.

### Pull-down assay

Aliquots of rat HOMO were diluted with TBS 1 × to a final volume of 1 ml and incubated 2 h with 26 μl of cold phosphorylated or not GST fusion proteins. After incubation, beads were washed five times with TBS and 0.1% Triton X-100. Bound proteins were resolved by SDS-PAGE and subjected to immunoblot analysis with anti-SAP97, anti-ADAM10, anti-AKAP150, anti-GluA1, anti-GluN2A and anti-GST Ab.

### Immunocytochemistry and colocalization analysis

Primary neurons were kept in culture 14 days *in vitro* (DIV) before treatment and staining experiments. For colocalization analysis, transfected neurons were fixed in 4% PFA with 4% sucrose in PBS (pH 7.4) at 4 °C or in methanol at −20 °C and immunostained for ADAM10, SAP97 and GM130 or PSD-95; primary and secondary antibodies were applied in GDB buffer^35^ (30 mM phosphate buffer, pH 7.4, containing 0.2% gelatin, 0.5% Triton X-100 and 0.8 M NaCl). To evaluate surface and total staining, transfected COS-7 cells were fixed with 4% PFA, 4% sucrose in PBS (pH 7.4) and then incubated with anti-ADAM10 Ab. To visualize surface expression, cells were then blocked with 4% normal serum, followed by a 555-conjugated secondary Ab. Afterwards, cells were permeabilized with 0.1% Triton X-100 for 10 min and intracellular expression of ADAM10 and SAP97 was determined by incubating cells with the appropriate Ab, anti-ADAM10 Ab or anti-green fluorescent protein (GFP) Ab (for SAP97wt and mutants constructs), and then with a 488- or 633-conjugated secondary Ab.

### *In vitro* fusion protein phosphorylation assay

GST-purified fusion proteins were incubated or not with 10 ng of active PKC for 30 min at 37 °C, in the presence of 10 mM Tris-HCl (pH 7.4), 10 mM MgCl_2_, 0.5 mM CaCl_2_ and 10 *μ*M ATP ([*γ*-^32^P]ATP 2 *μ*Ci per tube; 3000 Ci/mmol; Perkin-Elmer, Waltham, MA, USA). Proteins were separated on SDS-PAGE and phosphoproteins were revealed by autoradiograph.

### Microscope image acquisition and data analysis

Fluorescence images were acquired using the AIM 4.2 software (Zeiss, Jena, Germany) and the confocal LSM510 Meta system (Zeiss) with a × 63 objective and a sequential acquisition setting at 1024 × 1024 pixel resolution; for each image, 0.5 *μ*m sections were acquired to span the entire cell and z-projection was obtained using the AIM 4.2 software. Images were processed using ImageJ (National Institute of Health, Bethesda, MD, USA) and Adobe Photoshop software (Adobe Systems Incorporated, San Jose, CA, USA). Quantification analyses were performed blind after randomization of the images. Quantification of WB analysis was performed by means of computer-assisted imaging (ImageJ) after normalization on tubulin or GST levels. Values are expressed as mean±S.E.M. of at least three independent experiments. For surface/total ratio assays and colocalization analysis, cells were chosen randomly for quantification from four different coverslips (two or three independent experiments), images were acquired using the same settings/exposure times and at least eight cells for each condition were analyzed. Colocalization analysis was performed using AIM 4.2 software (Zeiss) on a pixel basis. We draw an ROI of the area of interest and we evaluated the colocalization coefficients and expressed it as the percentage of colocalization. For quantification of surface/total ratios, all images were analyzed using Image J. The average intensity of surface fluorescence staining was determined after cell tracing, and normalized to the total intensity to correct for differences in expression. Surface ratios were obtained by dividing the background subtracted fluorescence intensities.

### Statistics

Statistical evaluations were performed by using two-tailed Student's *t*-test (a *P*-value <0.05 was considered significant) or, when appropriate, by using one-way ANOVA followed by Bonferroni's *post hoc* test.

### Study approval

All experimental procedures were carried out with care to minimize discomfort and pain to treated animals, in accordance with the guidelines of the European Communities Council (Directive of November 24, 1986, 86/609/EEC). For the experiments carried on human brain samples, all procedures were in accordance with the NIH Guide for Care and Use of laboratory human tissues and were approved by the Ethics Committee of the University of Milan. For the genetic analysis, written informed consent (from the subject or from the responsible guardian if the subject was incapable) was obtained, before study initiation, for blood collection and genotyping. The work conformed to the Declaration of Helsinki and approved by the local Ethics Committee.

## Abbreviations

A: alanine
AD: Alzheimer's disease
ADAM10: A disintegrin and metalloproteinase 10
AKAP: A-kinase anchor protein
APP: Amyloid Precursor Protein
BFA: brefeldin-A
BRET: bioluminescence resonance energy transfer
Ct: C terminal
CTRL: control
ER: endoplasmic reticulum
GFP: green fluorescent protein
GM130: *cis*-Golgi marker 130
GST: glutathione *S*-transferase
HC: healthy control
HOMO: homogenate
IP: immunoprecipitation
^32^P: phosphorus-32
PDBu: phorbol 12,13-dibutyrate
PKC: protein kinase C
PSD-95: postsynaptic density-95
P3: microsomal fraction
S: serine
SH3: SRC homology 3
T: threonine
TIF: Triton-insoluble fraction
Wt: wild-type
YFP: yellow fluorescent protein
