# Supplementary material for: SAP97-mediated ADAM10 trafficking from Golgi outposts depends on PKC phosphorylation
Source: Cell Death Dis. 2014 Nov 27;5(11):e1547–. doi: 10.1038/cddis.2014.492 (PMC4260750; doi:10.1038/cddis.2014.492)
Supplement: Supplementary Material and Methods [file cddis2014492x1.docx]

**Supplementary material and methods**

**Antibodies, reagents and cell-permeable peptides**. The following antibodies (Ab) were used: monoclonal (mAb) 22C11 (raised against N-terminal domain of APP, 66-81 aa), mAb 6E10 (raised against 1-17 aa of Aβ), polyclonal (pAb) anti-AMPA GluA1 and chicken pAb anti-GFP (Millipore, Billenca, MA, USA); pAb anti-ADAM10 (Abcam, Cambridge, MA, USA); mAb anti-ADAM10 (R&D, Minneapolis, MN, USA); pAb anti-AKAP150 (Santa Cruz Biotechnology, Dallas, Texas, USA); rabbit pAb anti-GFP (Synaptic System, Goettingen, Germany); mAb anti-GST, anti-SAP102 and anti-PSD-95 (NeuroMab, University of California Davis, Davis, CA, USA); mAb anti-SAP97 (Stressgen, Victoria, Canada); pAb anti-SAP97 and mAb DSI (ABR-Thermoscientific, Rockford, IL USA); mAb anti-α-Tubulin (Sigma-Aldrich, St. Louis, MO); mAb against GluN2A (Zymed, San Francisco, CA, USA); mAb anti-GM130 (BD Biosciences, NJ, USA). The SAP97-T629P phosphoantibody was raised against the synthetic peptide RQVTPDGESDE, corresponding to aa 626-636 of SAP97 where T629 is present. SAP97-T629P antibody was afﬁnity puriﬁed from sera by afﬁnity chromatography. Primm (Milan, Italy) performed synthetic peptides' synthesis, immunization and puriﬁcation. Peroxidase-conjugated secondary anti-mouse Ab (Pierce, Rockford, IL, USA); peroxidase-conjugated secondary anti-rabbit Ab (Bio-Rad, Hercules, CA, USA). AlexaFluor secondary Abs were purchased from Life Technologies (Carlsbad, CA). Phorbol 12,13-dibutyrate (PDBu), phorbol 12-myristate 13-acetate (PMA), Brefeldin-A (BFA) and ATP were purchased from Sigma-Aldrich; PKC and PKC lipid activator from Millipore; [γ-^32^P] ATP and [^32^P] orthophosphate from PerkinElmer, USA. The cell permeable Pro peptide was obtained linking the 11 amino acids human immunodeficiency virus Tat transporter sequence [^1^](#_ENREF_1) to the 21 amino acids sequence corresponding to ADAM10 proline-rich domains (NH2-YGRKKRRQRRR-PKLPPPKPLPGTLKRRRPPQP-COOH). Ala peptide, in which all proline residues were substituted by alanine (NH2-YGRKKRRQRRR-AKLAAAKALAGTLKRRRAAQA-COOH), was used as the control [^2^](#_ENREF_2). All peptides were synthesized by Bachem (Switzerland).

**DNA constructs.** The mouse ADAM10 cDNA was provided by from Dr. Saftig (Biochemical Institute, Christian-Albrechts-University Kiel, Germany). The plasmid encoding GST-SAP97wt was kindly provided by Kari Keinänen (Helsinki, Finland). The point mutations (GST-SAP97-S582A, GST-SAP97-S597A, GST-SAP97-T629A, GST-SAP97-S642A) and the deletion (GST-SAP97-624Δ and GST-SAP97-GKΔ) were generated by site-direct mutagenesis. The plasmid encoding SAP97wt (SAP97-YFP-Nterm i3) was kindly provided by Mark L. Dell'Acqua (Aurora, Colorado, USA). The mutants YFP-SAP97-T629A, YFP-SAP97-T629D, YFP-SAP97-S642A and YFP-SAP97-S642D were created from YFP-SAP97wt by site-direct mutagenesis. For BRET experiments, the coding region of ADAM10 was subcloned in RLuc (*Renilla luciferase*) plasmid in-frame with the 5' end of Rluc to obtain ADAM10-Rluc. All constructs were verified by sequencing.

**Acute hippocampal slices, cell cultures, transfection and treatments.** Acute hippocampal slices were prepared as previously described [^3^](#_ENREF_3)^,^ [^4^](#_ENREF_4). Hippocampal neuronal cultures were prepared from E18-E19 rat hippocampi as previously described [^5^](#_ENREF_5). Neurons were transfected using the calcium phosphate precipitation method at 10 days in vitro (DIV). COS-7 cells, HEK293 cells and HEK293 cells stably transfected with human APP were transiently transfected using Lipofectamine LTX (Invitrogen) and were grown for 24 or 48 h before analysis.

Slices and neuronal culture at DIV14 were treated with either PDBu 100 nM or Ala or Pro peptides 1 µM for 30 min, or pre-treated with either Ala or Pro peptides 1 µM or BFA 10 µg/ml and then treated with PDBu 100 nM.

**SDS PAGE.** Samples were separated by electrophoresis on SDS–PAGE gel, transferred to a nitrocellulose membrane and probed with the corresponding primary Ab, followed by incubation with horseradish peroxidase-conjugated secondary. Membranes were developed using electrochemiluminescence reagent (Bio-Rad).

**BRET.** For total fluorescence and luminescence measurements, HEK293 cells were dispersed in 96-well plates with clear bottoms (Costar) at a density of 100,000 cells/well. We measured the total fluorescence of the cells in a Mithras LB 940 instrument (Berthold Technologies) using an excitation filter at 485 nm and an emission filter at 530 nm. The light emitted at 530 nm upon light excitation at 485 nm (fluorescence) is indicative of the amount of YFP-tagged proteins. We then incubated cells with DeepBlueC coelenterazine (5 μM) to measure the total luminescence using the Mithras instrument. The light emitted at 400 nm in the presence of DeepBlue C (luminescence) is indicative of the amount of Rluc-tagged proteins. For BRET measurements, cells coexpressing ADAM10-Rluc and YFP-SAP97 constructs were distributed in white 96-well microplates (Corning) at a density of 100,000 cells/well. BRET was then monitored by successive reading in the 510–550 nm (YFP) and 460–500 nm (Rluc) using nm windows using filters with the appropriate band pass immediately after addition of Coelenterazine H in PBS (5 µM final) in the Mithras instrument. The BRET signal is determined by calculating the ratio of the light emitted by the YFP over the light emitted by the Rluc. Each condition was loaded in triplicate wells and values were corrected by subtracting the background ratio detected when the Rluc construct was expressed alone. To evaluate PKC activation effect, PMA 100 nM was added and BRET measured for 30 min.

**SAP97 exon17 sequencing.** Genomic DNA will be prepared from 2.7 ml blood using the salting out method. SAP97 exon17 and 157 bp of its flanking introns were evaluated by PCR. PCR primers were designed to optimize denaturing dHPLC conditions (forward primer: 5’- TCAGAACCCTATGATATGTTGAT -3’ and reverse primer: 5’- CATCCATGTTGTCACAAATTAAA -3’) with the annealing temperature of 54°C. PCR was performed with twenty nanograms of genomic DNA. The cycling programme included an initial denaturation at 95°C for 2 min, followed by 40 cycles of denaturation at 95°C for 30s, annealing at 54°C for 30s, extension at 72°c for 30s and a final 5 min extension at 72°C. The PCR products were separated on a 2% agarose gel and the fragments were evaluated by dHPLC analysis (Transgenomic, Santa Clara, CA), and samples with an altered dHPLC profile were sequenced. Nucleotide direct sequencing was performed on genomic DNA for both strands by ABI 3130xl DNA analyzer (Applied Biosystems, Foster City, CA, USA), and analyzed using SeqScape Software version 2.6 (Applied Biosystems, Foster City, CA, USA).

**Metabolic labeling.** COS-7 cells were transfected with SAP97-YFP. 48 h after transfection, cells were preincubated for 1 h in phosphate-free MEM. The medium was replaced with fresh phosphate-free MEM containing [^32^P]orthophosphate (500 µCi/ml). After 1h PDBu 100 nM was added to the medium, and cells were incubated for 1h. Cells were harvested into ice-cold solubilization buffer (50 mM Hepes, 0,5 M NaCl, 0,5% Triton, 1 mM EDTA, 0,1% DOC) containing a complete set of protease inhibitors. Cell lysates were immunoprecipitated with anti-GFP Ab, run on 7% SDS-PAGE, and exposed for analysis.

**Modelling studies.** The phosphorylation of residue T629 was modelled in the 3D structure of SAP97 (PDB code:3UAT) [^6^](#_ENREF_6) using the tleap module of Amber 12 [^7^](#_ENREF_7). The structure of the SH3 domain in absence and presence of the phosphorylation was placed in a box filled with TIP3P water molecules [^8^](#_ENREF_8) and made electroneutral by the addition of 8 and 9 Na+ counterions, respectively. The final systems consist of ~ 27000 atoms. The topologies of the systems were obtained using the ff99bsc0 Amber force field. The systems were subjected to 5000 steps of minimization with the steepest descent algorithm.

The systems were equilibrated in the NPT ensemble (constant moles (N), pressure (P) and temperature (T) with runs of 200 ps of minimization each at the temperatures of 50, 150, 200, 250 K. After equilibration, the systems were subjected to two simulations of 10 ns in the NVT ensemble (constant moles (N), volume (V) and temperature (T) using Amber 12 for GPU [^9^](#_ENREF_9).

The electrostatic potential was calculated using the APBS [^10^](#_ENREF_10) plug-in implemented in the VMD v. 1.9.1 program [^11^](#_ENREF_11).

The structures of the SH3 domains used for the superposition with the SAP97 domain were selected based on two characteristics: 1) resolution better than 2 Å; 2) presence of a co-crystallized peptide. The selected domains are (PDB code): 1BBZ, 1CKA, 1SEM, 1UTI, 1ZUK, 2D1X, 2GF6, 2J6F, 2O88, 2O9V, 2P4R, 2W0Z, 3I5R, 4EIK and 2KXC.

The 15 domains were superimposed to the structure of the SAP97 SH3 phosphorylated domain after the simulation. The superposition was performed using the UCSF Chimera 1.6 program [^12^](#_ENREF_12).

**References**

1. Aarts M, Liu Y, Liu L, Besshoh S, Arundine M, Gurd JW*, et al.* Treatment of ischemic brain damage by perturbing NMDA receptor- PSD-95 protein interactions. *Science* 2002, **298**(5594)**:** 846-850.

2. Marcello E, Gardoni F, Mauceri D, Romorini S, Jeromin A, Epis R*, et al.* Synapse-associated protein-97 mediates alpha-secretase ADAM10 trafficking and promotes its activity. *J Neurosci* 2007, **27**(7)**:** 1682-1691.

3. Gardoni F, Bellone C, Cattabeni F, Di Luca M. Protein kinase C activation modulates alpha-calmodulin kinase II binding to NR2A subunit of N-methyl-D-aspartate receptor complex. *J Biol Chem* 2001, **276**(10)**:** 7609-7613.

4. Gardoni F, Mauceri D, Fiorentini C, Bellone C, Missale C, Cattabeni F*, et al.* CaMKII-dependent phosphorylation regulates SAP97/NR2A interaction. *J Biol Chem* 2003, **278**(45)**:** 44745-44752.

5. Gardoni F, Bellone C, Viviani B, Marinovich M, Meli E, Pellegrini-Giampietro DE*, et al.* Lack of PSD-95 drives hippocampal neuronal cell death through activation of an alpha CaMKII transduction pathway. *Eur J Neurosci* 2002, **16**(5)**:** 777-786.

6. Zhu J, Shang Y, Xia C, Wang W, Wen W, Zhang M. Guanylate kinase domains of the MAGUK family scaffold proteins as specific phospho-protein-binding modules. *EMBO J* 2011, **30**(24)**:** 4986-4997.

7. Case DA, Cheatham TE, 3rd, Darden T, Gohlke H, Luo R, Merz KM, Jr.*, et al.* The Amber biomolecular simulation programs. *Journal of computational chemistry* 2005, **26**(16)**:** 1668-1688.

8. Jorgensen WL, Chandrasekhar J, Madura JD, Impey RW, Klein ML. Comparison of simple potential functions for simulating liquid water. *The Journal of chemical physics* 1983, **79**(2)**:** 926-935.

9. Gotz AW, Williamson MJ, Xu D, Poole D, Le Grand S, Walker RC. Routine Microsecond Molecular Dynamics Simulations with AMBER on GPUs. 1. Generalized Born. *Journal of chemical theory and computation* 2012, **8**(5)**:** 1542-1555.

10. Baker NA, Sept D, Joseph S, Holst MJ, McCammon JA. Electrostatics of nanosystems: application to microtubules and the ribosome. *Proc Natl Acad Sci U S A* 2001, **98**(18)**:** 10037-10041.

11. Humphrey W, Dalke A, Schulten K. VMD: visual molecular dynamics. *Journal of molecular graphics* 1996, **14**(1)**:** 33-38, 27-38.

12. Pettersen EF, Goddard TD, Huang CC, Couch GS, Greenblatt DM, Meng EC*, et al.* UCSF Chimera--a visualization system for exploratory research and analysis. *Journal of computational chemistry* 2004, **25**(13)**:** 1605-1612.
