# Supplementary figures and images for: SAP97-mediated ADAM10 trafficking from Golgi outposts depends on PKC phosphorylation
Source: Cell Death Dis. 2014 Nov 27;5(11):e1547–. doi: 10.1038/cddis.2014.492 (PMC4260750; doi:10.1038/cddis.2014.492)

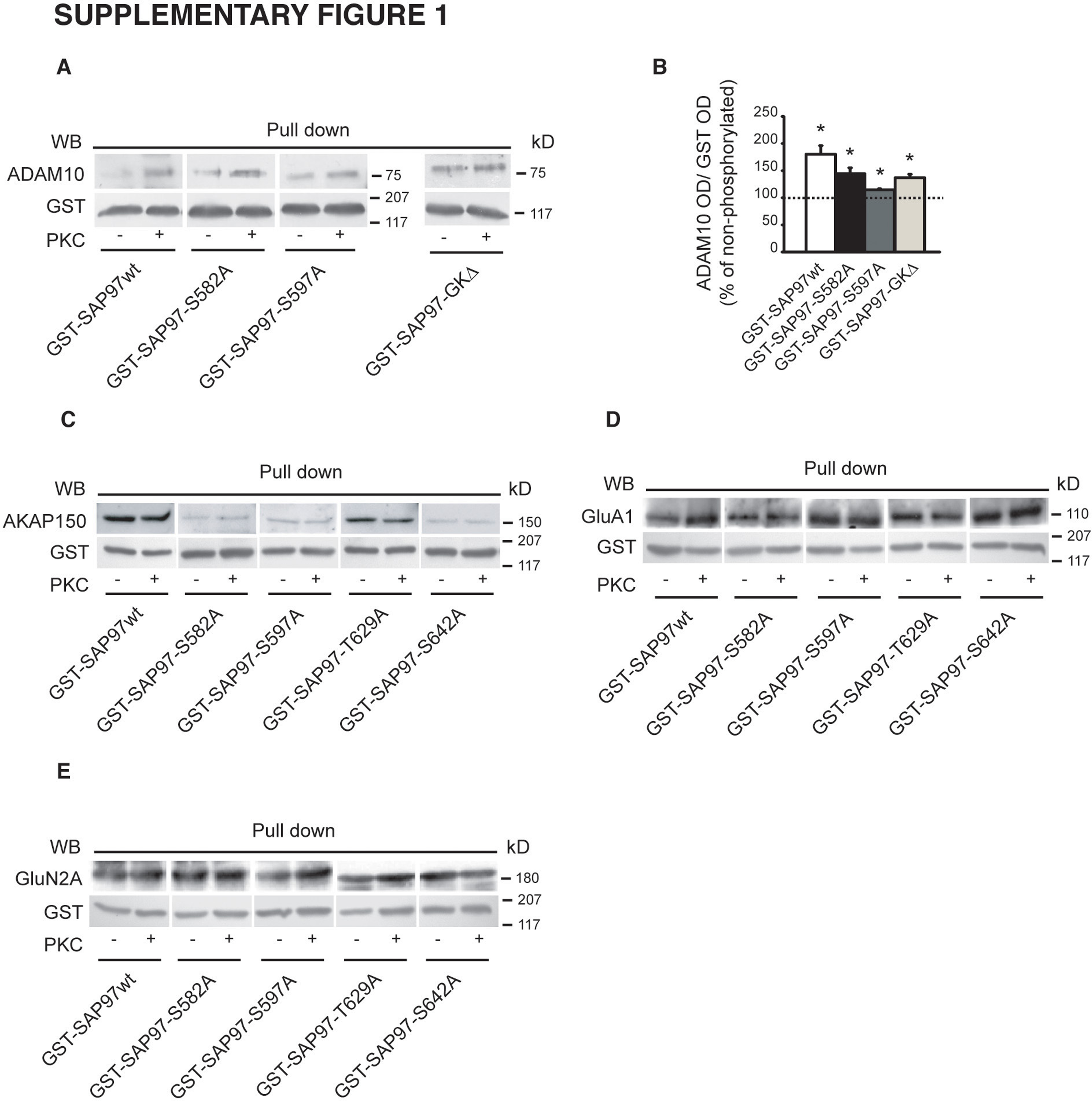

Supplement: Supplementary Figure 1 [file cddis2014492x2.tif]

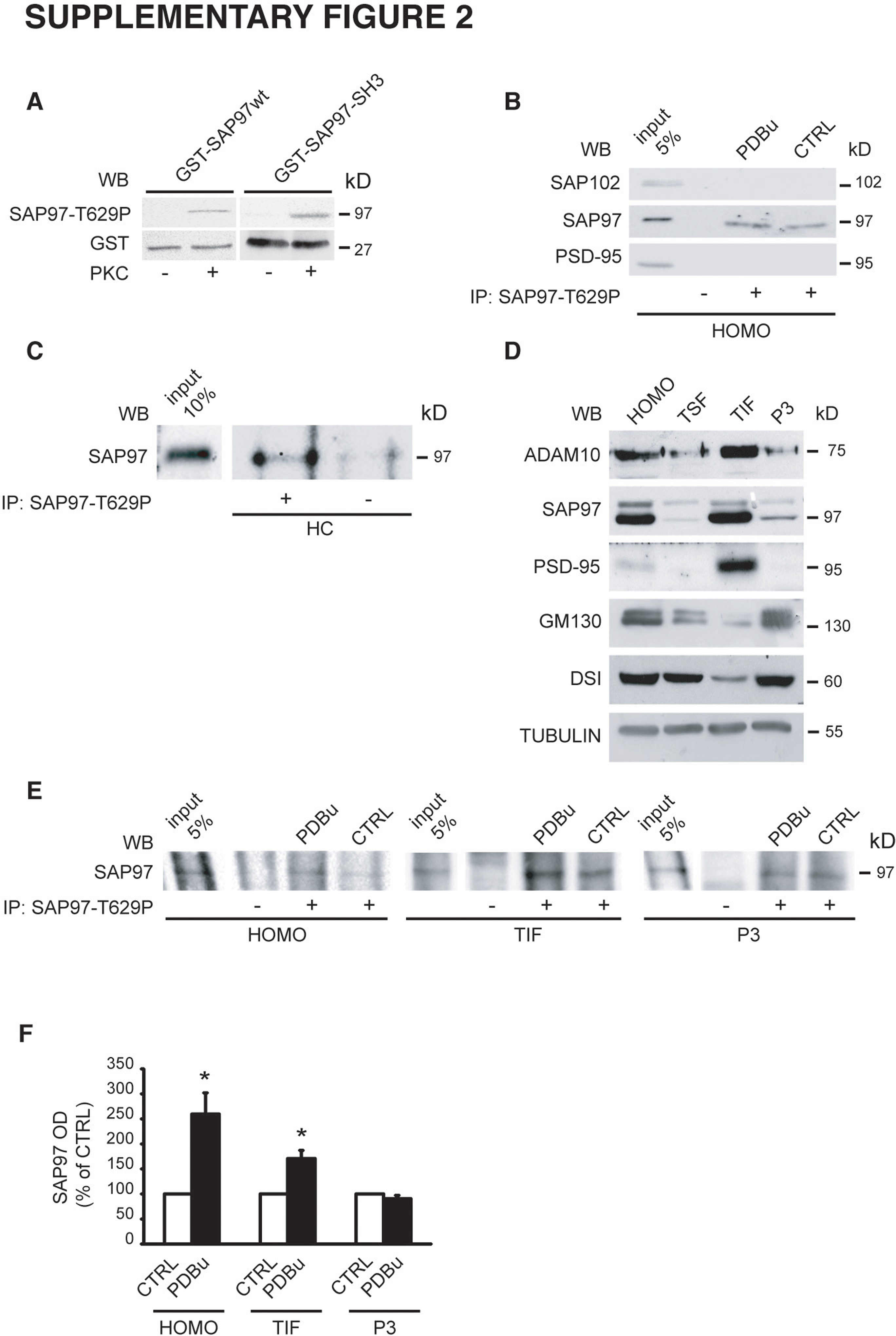

Supplement: Supplementary Figure 2 [file cddis2014492x3.tif]

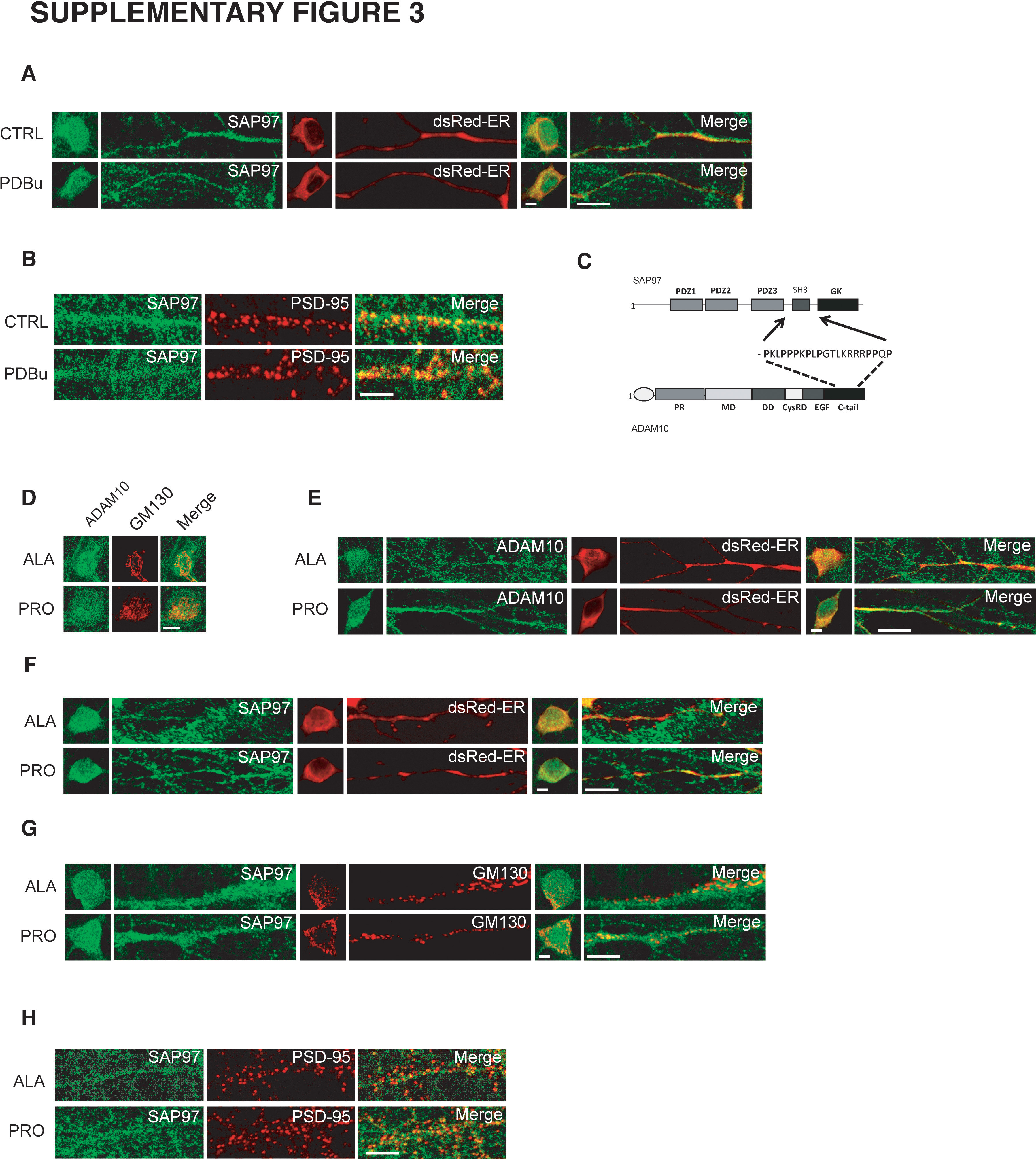

Supplement: Supplementary Figure 3 [file cddis2014492x4.tif]

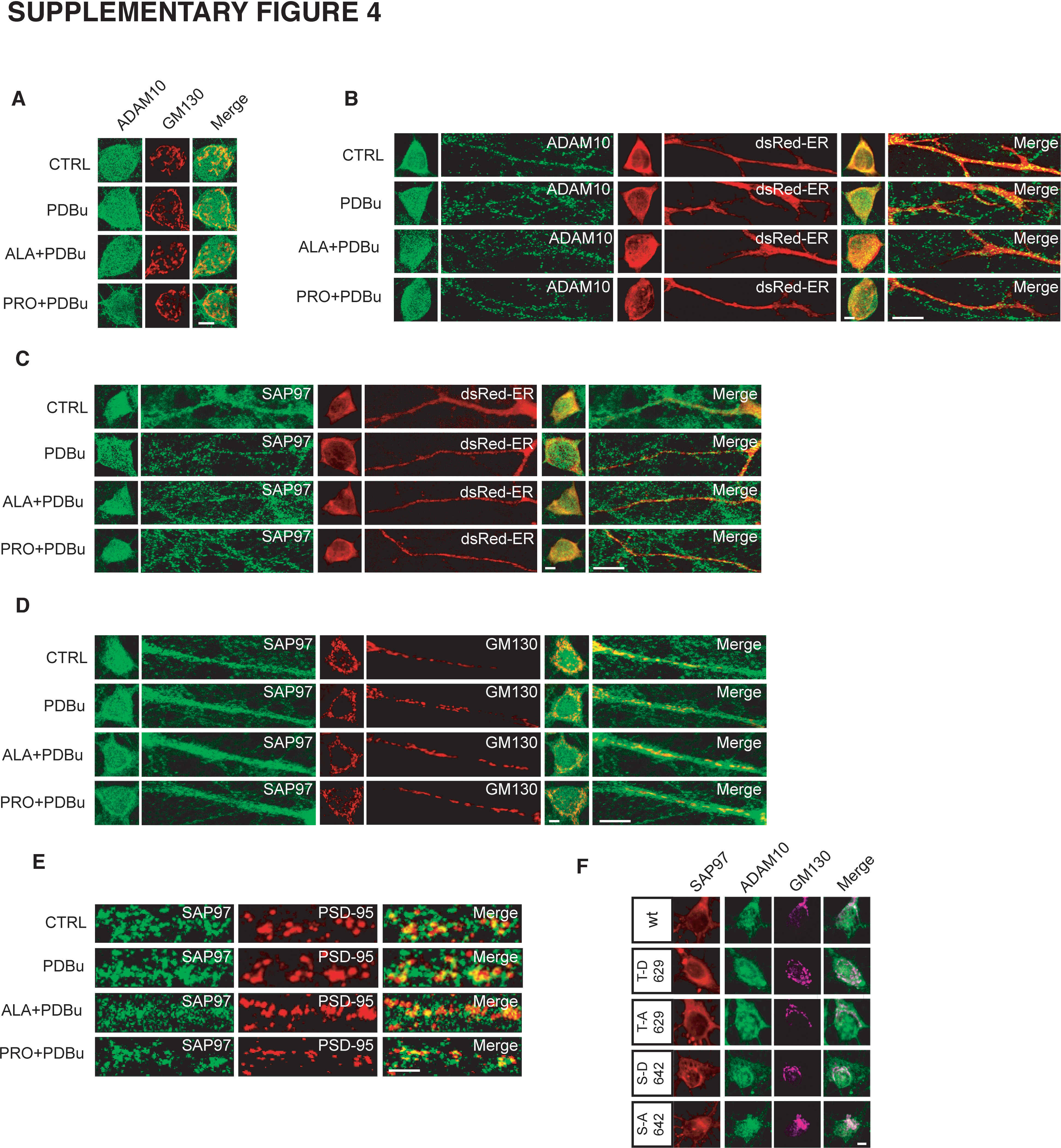

Supplement: Supplementary Figure 4 [file cddis2014492x5.tif]
