# Supplementary material for: SAP97-mediated ADAM10 trafficking from Golgi outposts depends on PKC phosphorylation
Source: Cell Death Dis. 2014 Nov 27;5(11):e1547–. doi: 10.1038/cddis.2014.492 (PMC4260750; doi:10.1038/cddis.2014.492)
Supplement: Supplementary Figure Legends [file cddis2014492x6.docx]

**Supplementary figures legends**

**Supplementary Figure 1 - Analysis of the effect of lack of SAP97 phosphosites on the binding to ADAM10, AKAP150, GluA1 and GluN2A.**

**A** GST-SAP97wt and different mutants were PKC-phosphorylated and then incubated with rat brain homogenate to carry out a pull-down assay. WB analysis was performed using anti-ADAM10 and GST antibodies. The lack of these phosphosites does not affect the PKC-induced increase in ADAM10 binding to SAP97.

**B** Quantification of experiments in A (*n*=3, * *P*=0.03 GST-SAP97wt phosphorylated *vs* non-phosphorylated; *P*=0.05 GST-SAP97-S582A phosphorylated *vs* non-phosphorylated; *P*=0.015 GST-SAP97-S597A phosphorylated *vs* non-phosphorylated; *P*=0.03 GST-SAP97-GKΔ phosphorylated *vs* non-phosphorylated; paired *t*-test).

**C, D, E** GST-SAP97wt and different mutants were incubated with rat brain homogenate after PKC phosphorylation to analyze AKAP150 (C), GluA1 (D) and GluN2A (E) precipitation in a pull down assay (AKAP150: GST-SAP97wt = -0.2 ± 2.7%; GST-SAP97-S582A = -11.1 ± 5.6%; GST-SAP97-S597A = +17.6 ± 3.2%; GST-SAP97-T629A = +6.8 ± 5.2%; GST-SAP97-S642A = +8.9 ± 7.7%; GluA1: GST-SAP97wt = +0.9 ± 9.3%; GST-SAP97-S582A = +0.7 ± 5.9%; GST-SAP97-S597A = +19.2 ± 7%; GST-SAP97-T629A = +22.2 ± 6.7%; GST-SAP97-S642A = +6.3 ± 12%; GluN2A: GST-SAP97wt = -15.7 ± 7.3%; GST-SAP97-S582A = -14.8 ± 4.7%; GST-SAP97-S597A = -8 ± 8%; GST-SAP97-T629A = -3.6 ± 3.1%; GST-SAP97-S642A = +4 ± 9.6%; *n*=3, *P*>0.05, phosphorylated *vs* non- phosphorylated, paired *t*-test).

**Supplementary Figure 2 - Validation of the** **SAP97-T629P Ab recognizing SAP97 phosphorylated at T629.**

**A** GST-SAP97wt and GST-SAP97-SH3 were incubated or not with PKC in the presence of cold ATP. WB analysis performed with SAP97-T629P Ab revealed that the antibody is able to recognize only the phosphorylated proteins.

**B** HOMO aliquots of CTRL and PDBu-treated hippocampal slices were IP with SAP97-T629P Ab and the WB was carried out with Ab recognizing SAP97, SAP102 and PSD-95. The phosphoantibody SAP97-T629P is able to precipitate only SAP97 but not SAP102 and PSD-95.

**C** HOMO aliquots of HC hippocampus was IP with anti-SAP97-T629P and total SAP97 was evaluated by WB analysis. The IP was specific because no signal was detected in the absence of the phosphoantibody.

**D** Representative WB of ADAM10, SAP97 and different protein markers. Homogenate (HOMO), Triton Soluble Fraction (TSF), Triton Insoluble Fraction (TIF), and microsomal fraction (P3). Markers used to evaluate fraction enrichment: GM130, Golgi resident protein, DSI as marker of ER, PSD-95, PSD marker.

**E** 70 µg of homogenate, 5 µg of TIF and 30 µg of P3 of CTRL and PDBu-treated hippocampal slices were IP with SAP97-T629P and WB was carried out with anti-SAP97 antibody.

**F** Quantification of IP experiment in E (*n*=3, * *P*=0.03*,* HOMO, PDBu *vs* CTRL; *P*=0.01, TIF, PDBu *vs* CTRL, paired *t*-test).

**Supplementary Figure 3 - ADAM10 and SAP97 localization in neurons treated with PDBu or incubated with Ala/Pro peptides.**

**A** Representative staining of SAP97/dsRed-ER in dendrites and soma of hippocampal neurons after PDBu treatment (SAP97/dsRed-ER: dendrites= -9.7 ± 4.7%, soma = -6.8 ± 3.6%; 24 neurons per condition from 3 independent experiments, *P*>0.05, unpaired *t*-test). Scale bar, 10 µm.

**B** SAP97/PSD-95 staining upon PKC activation (SAP97/PSD-95 = +12 ± 1.8%; 24 neurons per condition from 3 independent experiments, *P*>0.05, unpaired *t*-test). Scale bar, 10 µm.

**C** Schematic representation of SAP97 SH3 domain, ADAM10 Ct domain and of the aa sequence of the cell-permeable Pro peptide.

**D** Representative immunostaining of ADAM10 localization in somatic Golgi of hippocampal neurons after either Ala or Pro peptide treatment (ADAM10/GM130 = -0.6 ± 2.6%; 24 neurons per condition from 3 independent experiments, *P*>0.05, unpaired *t*-test). Scale bar, 10 µm.

**E** Representative ADAM10 immunostaining in somatic and dendritic ER of either Ala or Pro treated hippocampal neurons (ADAM10/dsRed-ER: dendrites = -13.2 ± 5.6%; soma = -2 ± 6%; 24 neurons per condition from 3 independent experiments, *P*>0.05, unpaired *t*-test). Scale bar, 10 µm.

**F, G, H** Representative immunostaining of SAP97 localization in somatic and dendritic ER (F), in somatic and dendritic Golgi (G) and in the PSD (H) of either Ala or Pro peptide-treated neurons (SAP97/dsRed-ER: dendrites = -19.2 ± 2.8%; soma = +1.5 ± 4.5%; SAP97/GM130: dendrites = +13.7 ± 3.3%; soma = +20 ± 1.6%; SAP97/PSD-95: = +0.3 ± 1.2%; 24 neurons per condition from 3 independent experiments, *P*>0.05, unpaired *t*-test). Scale bar, 10 µM.

**Supplementary Figure 4 - Analysis of ADAM10 and SAP97 localization in neurons pre-incubated with Ala/Pro peptides and treated with PDBu and of phosphomimetic mutants of SAP97.**

**A** ADAM10/GM130 staining in soma of hippocampal neurons pre-treated with either Ala or Pro peptide and exposed to PDBu (ADAM10/GM130, soma: PDBu = -25.7 ± 1.6%; ALA+PDBu = -23.7 ± 1.4%; PRO+PDBu = -26.9 ± 1.1%; 24 neurons per condition from 3 independent experiments, *P*>0.05, PDBu, ALA+PDBu and PRO+PDBu *vs* CTRL, one-way ANOVA, Bonferroni's post hoc test). Scale bar, 10 µm.

**B** Representative immunostaining of ADAM10/dsRed-ER in dendrites and soma of Ala/Pro peptide-treated hippocampal neurons and then incubated with PDBu (ADAM10/dsRed-ER, dendrites: PDBu = -21.3 ± 4.7%; ALA+PDBu = -21.8 ± 4.8%; PRO+PDBu = -31.8 ± 4%; soma: PDBu = -26.6 ± 5.3%; ALA+PDBu = -24 ± 4.4%; PRO+PDBu = -37.3 ± 4.8%; 24 neurons per condition from 3 independent experiments, *P>*0.05, PDBu, ALA+PDBu and PRO+PDBu *vs* CTRL, one-way ANOVA, Bonferroni's post hoc test). Scale bar, 10 µm.

**C, D** Representative immunostaining of SAP97/dsRed-ER and SAP97/GM130 colocalization in dendrites and soma of hippocampal neurons pre-treated with either Ala or Pro peptide and exposed to PDBu (SAP97/dsRed-ER, dendrites: PDBu = -8.6 ± 3.2%; ALA+PDBu = -7.24 ± 1.5%; PRO+PDBu = -5.4 ± 1.5%; soma: PDBu = -7.3 ± 2.7%; ALA+PDBu = -7.1 ± 1.1%; PRO+PDBu = -3.6 ± 1.4%; 24 neurons per condition from 3 independent experiments, *P*>0.05; SAP97/GM130, dendrites: PDBu = -42.83 ± 1.2; ALA+PDBu = -40.5 ± 1.2%; PRO+PDBu = -41.1 ± 1.4%; soma: PDBu = -39.4 ± 0.8%; ALA+PDBu = -36.3 ± 0.9%; PRO+PDBu = -36.7 ± 0.8%; 24 neurons per condition from 3 independent experiments, *P>*0.05, PDBu, ALA+PDBu, PRO+PDBu *vs* CTRL, one-way ANOVA, Bonferroni's post hoc test). Scale bar, 10 µm.

**E** SAP97/PSD-95 immunostaining of Ala/Pro peptide pre-treated neurons exposed to PDBu. Scale bar, 10 µM. (SAP97/PSD-95: PDBu = +3.7 ± 2%; ALA+PDBu = +1.6 ± 1.3%; PRO+PDBu = -2.4 ± 0.7%; 24 neurons per condition from 3 independent experiments, *P*>0.05, one-way ANOVA, Bonferroni's post hoc test). Scale bar, 10 µm.

**F** Representative ADAM10 localization in the somatic Golgi of neurons expressing either SAP97wt or SAP97 phosphosites mutants (ADAM10/GM130, soma: YFP-SAP97-T629D = -0.28 ± 6.5%; YFP-SAP97-T629A = -2.5 ± 6.4%; YFP-SAP97-S642D = -2 ± 6.2%; YFP-SAP97-S642A = -1.8 ± 3.7%; 16 neurons per condition from 2 independent experiments, *P*>0.05, one-way ANOVA, Bonferroni's post hoc test). Scale bar, 5 µM.
